# Progress and promise in understanding the genetic basis of common diseases

**DOI:** 10.1098/rspb.2015.1684

**Published:** 2015-12-22

**Authors:** Alkes L. Price, Chris C. A. Spencer, Peter Donnelly

**Affiliations:** 1 Department of Epidemiology, Harvard T. H. Chan School of Public Health, 655 Huntington Ave, Boston, MA 02115, USA; 2 Department of Biostatistics, Harvard T. H. Chan School of Public Health, 655 Huntington Ave, Boston, MA 02115, USA; 3 The Wellcome Trust Centre for Human Genetics, University of Oxford, Roosevelt Drive, Oxford OX3 7BN, UK; 4 Department of Statistics, University of Oxford, 1 South Parks Road, Oxford OX1 3TG, UK

**Keywords:** common diseases, genome-wide association studies, human genetics

## Abstract

Susceptibility to common human diseases is influenced by both genetic and environmental factors. The explosive growth of genetic data, and the knowledge that it is generating, are transforming our biological understanding of these diseases. In this review, we describe the technological and analytical advances that have enabled genome-wide association studies to be successful in identifying a large number of genetic variants robustly associated with common disease. We examine the biological insights that these genetic associations are beginning to produce, from functional mechanisms involving individual genes to biological pathways linking associated genes, and the identification of functional annotations, some of which are cell-type-specific, enriched in disease associations. Although most efforts have focused on identifying and interpreting genetic variants that are irrefutably associated with disease, it is increasingly clear that—even at large sample sizes—these represent only the tip of the iceberg of genetic signal, motivating polygenic analyses that consider the effects of genetic variants throughout the genome, including modest effects that are not individually statistically significant. As data from an increasingly large number of diseases and traits are analysed, pleiotropic effects (defined as genetic loci affecting multiple phenotypes) can help integrate our biological understanding. Looking forward, the next generation of population-scale data resources, linking genomic information with health outcomes, will lead to another step-change in our ability to understand, and treat, common diseases.

## 1. Introduction

Genetics plays a role in susceptibility to all common human diseases and to many other complex human traits. The genetic variants an individual inherits are only part of the story of disease susceptibility—most common diseases are 30–60% heritable—with lifestyle and dietary factors, and other environmental exposures, also playing an important role.

The last decade has seen an explosion in our knowledge of genetic variants associated with common diseases. Critical to this has been the ability to measure genetic variation at hundreds of thousands of markers across the human genome, in large numbers of individuals. Genome-wide association studies (GWAS) exploited these technological developments in large case-control studies, with unprecedented success.

In this review, we provide the background to GWAS and describe their success in identifying specific variants associated with disease, in assessing the total contribution of common variants to disease susceptibility, and in revealing often surprising genetic links between diseases. Each GWAS association potentially provides novel biological insights into disease pathophysiology, and potentially into novel therapeutic targets. Understanding of the biology underpinning GWAS associations has lagged behind their discovery, but even the limited number of new and in many cases unexpected biological findings that have followed from GWAS make it clear that the potential benefits are huge.

Prior to the GWAS era, there were two main approaches to understanding the genetic basis of common diseases. In spite of extensive research efforts, neither proved particularly fruitful. First, linkage studies searched for large disease-predisposing haplotypes shared among related affected individuals, with limited success. Linkage analysis is not well powered to identify common variants of modest effect, thus it did not detect the findings of subsequent GWAS [1]. Second, candidate-gene association studies focused on a specific gene or region of the genome and assessed variation in that gene/region for association with disease. While there were some successes, notably in breast cancer, most studies were based on what we now know to be poor choices of candidate loci, were underpowered, and/or were susceptible to confounding, leading to false-positive findings that failed to replicate [2]. Thus, prior to 2005, common variants reliably associated with susceptibility to common disease were largely limited to large-effect loci, such as the MHC in autoimmune disease, APOE in Alzheimer's and other diseases, and the sickle mutation for malaria susceptibility.

## 2. Genome-wide association studies

The ability to assay genome-wide genetic variation in large numbers of individuals has transformed our knowledge of the genetic architecture of common human diseases [1]. Several developments over the last decade have led to a dramatic increase in our knowledge.

The realization that there are extensive local correlations (called ‘linkage disequilibrium’) between nearby variants in the human genome led to the International HapMap Project [3], a major collaborative effort to map patterns of genetic variation in several global population groups. Critically, the extent of linkage disequilibrium in human populations means that much of the common variation in the genome (by convention, ‘common’ refers to variants with minor allele frequency (MAF) > 5%) can be assessed by directly typing only a subset of variation. For example, a carefully chosen set of approximately 500 000 SNPs covers over 80% of common variation in populations of European ancestry [4].

Parallel technological developments in array technologies, in part prompted by the HapMap project, allowed assays for hundreds of thousands of single-nucleotide polymorphisms (SNPs) in a single experiment. These SNP arrays were game-changing. They allowed, for the first time, a systematic, genome-wide assessment of the role of common genetic variation in human disease.

**Figure 1.**
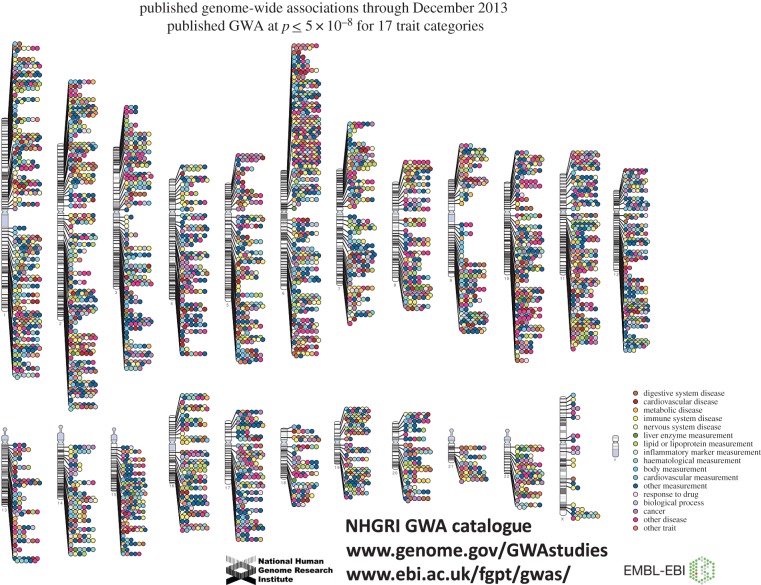
The NHGRI GWA catalogue. Published associations are displayed by chromosome and colour-coded by class of phenotype. As of December 2013, the catalogue included 11 912 SNPs that were significant at *p* < 10^−5^ and 6400 SNPs that were genome-wide significant (*p* < 5 × 10^−8^) [5]. Currently, the catalogue includes approximately 9400 genome-wide significant SNPs.

GWAS used SNP arrays to type hundreds of thousands of SNPs in large numbers of cases and controls for common diseases of interest. GWAS look for SNPs with statistically significant allele frequency differences between cases and controls. (The intuition is simple: if an allele increases susceptibility to a particular disease, that allele should be more common in cases than controls.) For quantitative traits, GWAS assess the correlation between SNP genotypes and trait values. The first GWAS were published in 2005, and within a few years there were over 100 DNA variants associated with disease susceptibility. There are now over 10 000 published associations, across hundreds of diseases and quantitative traits (figure 1; see also Web resources) [5].

To minimize false-positive findings, the field (or in practice its journal editors) have insisted on two criteria for declaring an association to be genuine. First, the association must meet stringent levels of statistical significance (e.g. *p* < 5 × 10^−8^). This has often been justified on the basis of multiple hypothesis testing [6], although others (including some of the current authors) have argued the multiple testing paradigm is not appropriate in the GWAS context (in brief, in assessing evidence for association with a particular SNP on chromosome 1, say, why should it matter whether one also looks at evidence for a completely different SNP on, say, chromosome 16?), but that stringent thresholds are nonetheless warranted due to the low prior probability of association (see box 1 in [7]). Second, the association must be replicated in a separate sample of cases and controls, ideally using a different genotyping technology to minimize the possibility of assay artefacts [8]. As a consequence of these criteria, along with extensive efforts to perform careful quality control and correct for confounding factors such as population stratification, the vast majority of published GWAS associations have proved to be real.

Most variants discovered by GWAS have relatively small effects on disease susceptibility, with typical odds ratios of 1.1 or lower, and rarely above 1.3. It has become clear (see below) that for a typical common human disease there will be a large number of SNPs that each have a small (but non-zero) effect on disease risk. Since power to detect an association depends on sample size and allele frequency, increasing the size of GWAS studies for a particular disease will lead to further discovery of associated variants. For relatively small GWAS size, one of the key factors driving success is the number of SNPs with common risk alleles at the top end of the GWAS range of effect size, which will differ across diseases. For example, early studies of age-related macular degeneration were successful with quite small sample sizes [9]. GWAS studies of a few thousand individuals have tended to yield reasonable numbers of associations for some autoimmune diseases, intermediate numbers of associations for diseases such as type 2 diabetes and heart disease, and small numbers (often none) for neuro-psychiatric disorders. At larger sample sizes, GWAS studies have been very successful, even in neuro-psychiatric diseases (e.g. a recent primary GWAS of 34 241 individuals with schizophrenia and 45 604 controls yielded 108 associations [10]).

The realization that sample size is a critical factor in GWAS success created strong incentives for groups studying a particular disease to collaborate by pooling their samples (e.g. by meta-analysing their association results). One positive side effect of the GWAS era is that there are now major global consortia in place to tackle the genetic basis of particular diseases, often involving tens of thousands of disease cases (or hundreds of thousands of individuals for quantitative traits); in some instances, the genotyping of large samples has been facilitated by cost-effective specialty chips targeting variants that were highly ranked in previous studies of related traits. Early in the GWAS era, there was debate about the relative merits of small, exquisitely phenotyped disease collections when compared with larger samples with limited or less precise phenotype information available. From a statistical perspective, this reduces to a question about noise and study power, and it has become clear that noise in the phenotype measurement can often be more than offset by large sample sizes. Even studies with self-reported phenotypes can be successful for at least some diseases [11].

**Figure 2.**
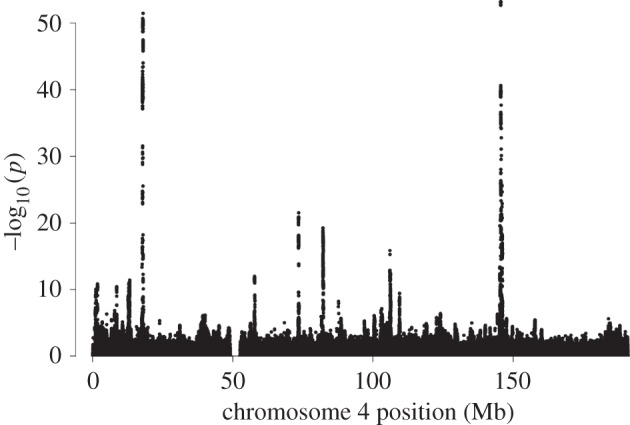
Manhattan plots from dense imputed data. A Manhattan plot for chromosome 4 for a GWAS of height in the UK Biobank imputed dataset of approximately 73 000 000 genetic variants (genome-wide) in approximately 150 000 individuals. The plot is adapted from fig. 3 in the document ‘UK Biobank phasing and imputation documentation’, written by J. Marchini (see Web resources).

One important methodological development for GWAS studies has been genotype imputation, introduced in 2007 [12]. Imputation further leverages the correlations between nearby alleles due to linkage disequilibrium. First, a relatively small sample of individuals (called a reference panel) is typed at a dense set of SNPs or directly sequenced. Then, given a much larger target sample of individuals typed at only a subset of the SNPs, knowledge of the correlation structure in the reference panel can be combined with the data from the target sample to predict, or impute, genotypes at untyped SNPs in the target sample. The accuracy of the imputed genotypes at untyped common variants is typically very high (e.g. *r*^2^ = 0.96 between imputed and true genotypes for common SNPs imputed using European reference samples from HapMap 3) [13]. Imputation accuracy decreases for low-frequency (0.5% < MAF < 5%) and rare (MAF < 0.5%) variants, but the latest imputation methods still perform well for many of these variants, and accuracy will increase as reference panels grow larger. The 1000 Genomes reference panel [14] is currently widely used, but larger reference panels such as the Haplotype Reference Consortium (see Web resources) are now available. In the context of GWAS, there have been two main uses of imputation. The first is to combine studies that use different genotyping arrays—SNPs typed in one study but not the other can be imputed in the other study to facilitate meta-analyses. The second is to use imputation from available reference panels to dramatically increase the number of variants for which genotype data are available for association testing (and fine-mapping; see below). Figure 2 illustrates part of the output from a GWAS study, called a Manhattan plot, in this case giving the strength of evidence of association of a phenotype with a dense set of imputed and directly genotyped SNPs.

Despite extensive study, relatively little is currently known about the underlying biological triggers and processes that lead to most diseases. Each GWAS association provides a potential clue about these mechanisms. If carrying one or two copies of a particular allele increases risk of a disease (relative to individuals not carrying that allele), then it should be possible to understand the different functional consequences, at a molecular and physiological level, of carrying that allele. In turn, this understanding can shed light on disease processes, and potentially point to novel targets for drug therapies. Progress in this harvesting of GWAS associations for novel biological insights has lagged considerably behind the rate of novel GWAS discovery. We discuss the reasons for this in the next section—it is a hard problem—and provide examples of some of the biological insights that have already arisen from GWAS findings. Assessment of the ultimate impact of GWAS discovery will be clearer when we have travelled further down the road of uncovering the underlying biology.

## 3. Biological insights from genome-wide association studies

It has become clear that changes in gene regulation, rather than changes to the proteins produced by genes, are at the heart of the biology underlying most GWAS associations (for example, top GWAS signals predominantly lie in intergenic and intronic regions). Owing to our lack of detailed knowledge of (and tools for studying) the biology of gene regulation, it has not been straightforward to move from GWAS findings to the underlying biological mechanisms, or even to definitive identification of the gene involved and direction of gene regulation effect. An added complication is that gene regulation effects are often tissue-specific, and for many diseases the appropriate tissue(s) or cell type(s) are not known precisely and/or are inaccessible and difficult to study. For example, for coronary artery disease, relevant tissue/cell types that could mediate genetic susceptibility include liver/hepatocytes (e.g. through involvement in lipoprotein metabolism, source of inflammatory cytokines), cells of the immune system (lymphocytes, macrophages), platelets and cells of the vessel wall, including smooth muscle cells and endothelial cells. Moreover, the biological mechanisms could operate at any stage of the individual's development and adult life (e.g. for neuro-psychiatric conditions, either brain development and/or adult brain could be important). For diseases involving the immune system (including but not exclusively autoimmune diseases) what is most relevant may be the way in which a specific immune cell type reacts to a particular external stimulus.

Although linkage disequilibrium is helpful at the stage of GWAS discovery (see above), it complicates GWAS follow-up: a GWAS discovery typically points to a region of linkage disequilibrium spanning tens of thousands of base pairs and tens of correlated SNPs, any of which could be driving the underlying molecular mechanism. GWAS results generally do not distinguish between the set of highly correlated SNPs. Further genetic study can be helpful, via fine-mapping, in which additional samples are typed at a denser set of SNPs in an effort to pinpoint causal variants. Fine-mapping is particularly effective when carried out in multiple ethnicities, because patterns of linkage disequilibrium (arising from shared ancestry) differ between populations. In a population different from the discovery sample, the biologically causal SNP may be in linkage disequilibrium with a different set of SNPs, thus reducing the overall set of potential proxy SNPs [15].

Despite these challenges, GWAS have produced valuable biological insights about the pathways involved in a particular disease, the functional mechanisms of (regulation of) a particular gene affecting the disease, and the functional and cell-type-specific enrichments for variants associated with the disease. Below, we discuss each of these in turn.

### (a) Biological pathways

Because much gene regulation acts in *cis*, biologically causal genes will often be physically close to the most associated GWAS SNP. GWAS associations are sometimes labelled by the closest gene, or by a nearby gene thought to be a good candidate for the disease or trait in question. This is often used in the field as a convenient shorthand in describing the association—it is not typically intended as a statement about the underlying biological basis for the association, nor should it be interpreted in this way. Nonetheless, the set of genes near a GWAS association is likely to be highly enriched for causal genes. One feature that became clear early in the GWAS era is that many of these potentially causal genes had not figured on the lists compiled by experts of likely candidate genes for the diseases in question, and hence that GWAS findings were pointing to genuinely novel biological insights for human disease.

Through a range of formal and informal analyses, examination of gene sets arising from GWAS findings have implicated specific and often novel pathways as being important for diseases studied. We illustrate just a few of many such examples. GWAS pointed to the previously unrealized importance of autophagy in Crohn's disease, and more generally has highlighted the importance of innate immune pathways in this condition [16]. In multiple sclerosis, GWAS confirmed a primary role for adaptive immunity, with surprisingly few neuronal hits [17], a finding reinforced by the overlap between GWAS variants for multiple sclerosis and those for other autoimmune diseases (see Pleiotropy section below for other instances of insights from comparisons of GWAS findings across diseases). In type 2 diabetes, the majority of GWAS loci appear to act primarily through defects in insulin secretion (implicating the pancreatic beta-cells) with unexpected pathways appearing to play key roles in disease pathogenesis, including cell cycle regulation and CREBBP-related transcription factor activity [18]. Parallel analyses of GWAS loci for glycaemic traits revealed the surprising finding that while many loci that affect levels of fasting glucose in non-diabetic individuals also affect risk of type 2 diabetes, some do not [19], indicating that the mechanism by which glucose homeostasis is disturbed, rather than simply the change in glucose levels, may be relevant to the pathophysiology of type 2 diabetes. Going forward, pathway analyses will continue to play a key role in interpretation of GWAS findings [20].

### (b) Functional mechanisms

Most GWAS loci contain common non-coding variants of modest effect whose signals span multiple genes. However, there are some examples where the top GWAS hit at a locus, or a SNP in strong linkage disequilibrium with it, is a protein-altering coding variant (e.g. the association at IL23R for Crohn's disease), and others where there is a second signal of association, independent of the main GWAS signal, caused by a protein-altering variant in one of the genes in the GWAS locus. These settings suggest both an obvious functional mechanism and gene of action. In other cases, loci may contain both regulatory variants for a particular gene and coding variants within that gene. For example, several GWAS loci for type 2 diabetes contain genes known to harbour causal coding variants for rare Mendelian syndromes related to type 2 diabetes (e.g. *HNF1A, HNF1B, WFS1, PPARG, KCNJ11, HNF4A, GCK*). In this case, a natural hypothesis is that the protein-coding change in each gene that causes the rare syndrome has a large effect on phenotype, whereas the (unknown) causal variant underlying the GWAS association affects regulation of the same gene and has a smaller effect on the disease. More broadly, conditional joint analyses of GWAS significant loci have often identified multiple independent signals at the locus [21].

GWAS findings and biological follow-up have suggested new treatment approaches for sickle cell disease (SCD), a substantial cause of mortality (176 000 deaths in 2013 [22]) and morbidity globally. SCD was first described over 100 years ago—and more than 60 years ago, it became arguably the first example of a disease where the underlying molecular basis was known—but in spite of intensive efforts, efficacious treatments for the disease have remained elusive. Although SCD is a Mendelian condition, novel biological insights arising from GWAS of a related trait have radically transformed the search for a treatment. Humans normally have several different types of haemoglobin. One type, haemoglobin F (HbF, fetal haemoglobin) predominates during fetal development and early life. At about six weeks of age, there is a switch (the ‘haemoglobin switch’) from HbF to haemoglobin A (HbA), which dominates throughout life. After this switch, there remain low levels of circulating HbF, with the exact amount differing between individuals. It had been observed that individuals with SCD (and also individuals with β-thalassemia) with higher levels of HbF suffer less severe symptoms, leading to treatments that aim to increase HbF levels. In parallel, there had been a long-standing, but largely unsuccessful interest in understanding the mechanism behind the haemoglobin switch, in the hope of fully or partially reversing it. Two GWAS treating levels of HbF as a quantitative trait identified the gene *BCL11A* [23,24], a transcription factor that had not previously been suggested as a player in the haemoglobin switch. A series of elegant studies have subsequently shown that production of HbF is controlled in a dosage-sensitive manner by BCL11A, and that inactivation of *BCL11A* in a mouse model for SCD corrects the defects associated with SCD by inducing HbF production [25], suggesting a natural focus for therapeutic intervention. It is worth noting that although SCD (and β-thalassemia) primarily affect individuals of non-European ancestry, the critical GWAS were undertaken in European ancestry groups. GWAS link genetic variation to phenotypes of interest, potentially leading to new biological understanding that will often be relevant across human populations, not just the population in which the GWAS was undertaken.

The 8q24 locus implicated in several common cancers provides another example of valuable biological insights. The locus was initially associated with prostate cancer, and later shown to harbour multiple independent variants influencing prostate cancer risk [26–28]. Subsequent studies showed that the locus is also associated with colorectal, breast, ovarian and bladder cancers [9,29,30]. Functional analyses have implicated a transcriptional enhancer involved in long-range interaction with the *MYC* oncogene and determined that mice lacking this enhancer are resistant to intestinal tumours [12,31].

A partial list summarizing some of the early successful approaches, and the resulting insights about functional mechanisms gained from GWAS, is provided in the electronic supplementary material, table S1.

A commonly expressed misconception about GWAS findings is that the small effect sizes at GWAS loci (which typically explain less than 1% of trait variance) are too small to be biologically interesting, or to be useful as drug targets. However, effect size and biological relevance are two distinct issues. Regardless of the effect size, each reliable association of genetic variation with a particular phenotype is the result of real biological mechanisms. Functional follow-up of the association can be used to reveal the mechanism, typically leading to new insights into human biology and disease pathophysiology, and potentially novel therapeutic targets [32]. By contrast, observed effect sizes for common genetic variants are a consequence of the interplay between the mutational events that give rise to genetic variation, and natural selection. If variants have large effects on disease susceptibility and on other phenotypes affecting fitness, then natural selection is likely to act to prevent those variants from becoming common. A drug targeting that gene may perturb the gene more than the GWAS variant does, and hence may have a larger effect on the downstream phenotype. Indeed there are several existing examples of exactly this. Statin drugs inhibit the enzyme HMGCo-A reductase. They reduce levels of circulating LDL cholesterol, and have had a substantial global impact on reducing cardiovascular disease. GWAS of LDL cholesterol levels found an association near the *HMGCo-A* reductase gene, but the GWAS variants had small effects [33]. Other examples of successful drugs targeting genes that arise (with small effect sizes) in GWAS include *PCSK9* for LDL cholesterol [33], and *PPARG* and *KCNJ11* for type 2 diabetes [18]. Each of these examples acts as a positive control for the hypothesis that GWAS variants of small effect can still point at effective drug targets.

### (c) Functional enrichment and cell-type-specific effects

Analyses of all GWAS loci (instead of a single GWAS locus) may also provide insights about functional mechanisms, by determining which functional annotations are enriched for association to disease. These analyses have largely focused on regulatory annotations using data generated by the ENCODE and Roadmap Epigenomics projects [34,35] to annotate predicted regulatory elements (e.g. enhancers), which are often cell-type-specific. One approach is to search for an enrichment of GWAS hits in one type of functional element seen in one cell type but not others. Several recent studies have reported that GWAS loci (and loci that do not reach genome-wide significance) exhibit cell-type-specific enrichments at DNase I hypersensitivity sites (DHS), histone marks and other regulatory elements. For example, variants associated with rheumatoid arthritis are enriched for H3K4me3 marks in CD4^+^ regulatory T cells [36], and variants associated with schizophrenia, body mass index and smoking behaviour are enriched for H3K27ac and other histone marks in brain tissues [10,37]. This information can also be used to prioritize causal variants, improving the efficacy of fine-mapping [38]. Most integrative analyses ultimately only provide suggestive hypotheses of causal mechanisms, with detailed functional follow-up being required to prove or support causality.

## 4. The polygenic architecture of common disease

GWAS have consistently demonstrated that most common diseases and traits are highly polygenic, with a large number of underlying genetic variants that affect the disease or trait [39]. As noted above, these common genetic variants generally have very small effects that require large sample sizes for detection [9] (with only a few exceptions). Furthermore, the set of associated variants often explain only a small proportion of the genetic variation in the disease or trait. For example, although the large schizophrenia study described earlier identified 108 associated loci, these loci collectively explain only 3% of the liability-scale variance of schizophrenia, a highly heritable trait [10]. As we have seen, the small effect sizes of associated variants do not preclude important biological insights, but the incomplete picture of the genetic architecture of diseases painted by the known associated loci has motivated intense efforts to explain the source of this ‘missing heritability’ [40].

An important step towards understanding the genetic architecture of common diseases, starting in 2010, was the use of statistical linear mixed models to estimate the heritability explained by all common variants, not just those associated at stringent statistical significance [41]. These studies showed that common variants typically explain roughly half of the narrow-sense heritability estimated from twin studies, with the remainder likely to be due to rare variants [42] (see below) and/or upward bias in twin-based estimates [43]. In other words much, or potentially even most, of the ‘missing heritability’ was not actually missing—it was explained by the many common variants whose effect sizes were small enough that they had not individually reached statistical significance for association. Quantifying the total number of common variants contributing non-zero effects remains an ongoing challenge, with initial estimates in the thousands for several traits [44]. Most studies of the polygenic architecture of common disease have focused on additive effects, as there is currently limited evidence of interactions between alleles within a locus (dominance) or between loci (epistasis).

## 5. Pleiotropy

**Figure 3.**
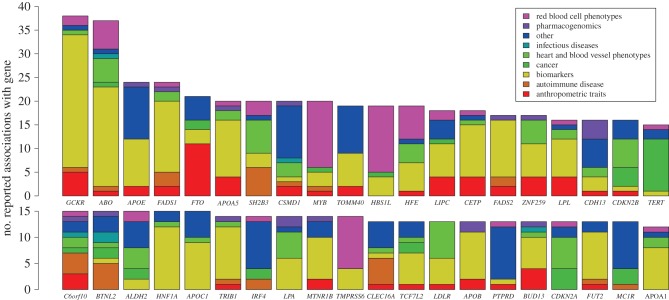
Genes involved in pleiotropy. Barplot of the 40 genes in the NHGRI GWAS catalogue (www.genome.gov/gwastudies, accessed 23 October 2014) that have the highest number of associations where they are listed as the reported gene. Genes in the MHC region have been excluded. Colours show the number of associations that are attributed to each different category of study phenotypes.

The term pleiotropy is used to describe the phenomenon whereby genetic variation at a single locus has an effect on more than one phenotype. A related but different concept is genetic correlation, defined as a correlation in direction and magnitude of genetic effects. (Genetic correlation is a specific measure of the extent to which genome-wide SNPs have the same direction and magnitude of effect on two phenotypes.) Some care is needed in differentiating different types of pleiotropy and genetic correlation in associations across traits [45], particularly when the causal SNPs have yet to be identified. When pleiotropy is observed, Mendelian randomization, which employs significantly associated variants as instrumental variables, can be used to assess causal relationships between risk factors and disease [46]. Some genes seem to be particular foci for association with multiple phenotypes (figure 3). These include genes whose function is at least partially understood, such as the gene *ABO* (which determines the ABO blood group), where a complex pattern of association is found with infectious diseases such as malaria, red cell counts, inflammatory, lipid and liver biomarkers, common cardiovascular disease and Grave's disease [5].

Autoimmune disease encompasses a range of disorders in which it is thought that immune and inflammatory mechanisms damage normal tissues in the body. GWAS have yielded hundreds of loci across the genome that are robustly associated with the risk of developing one or more of these disorders, and it is therefore one of the areas where pleiotropy has been studied most extensively. These analyses have shown that many susceptibility alleles are shared across autoimmune disorders [47,48]. Classifying shared associations according to whether the direction of effect is the same or different between phenotypes provides further evidence about the molecular relationship between diseases [49]. One example is a putative loss of function variant in *PTPN22* that decreases the risk of Crohn's disease but increases the risk of rheumatoid arthritis and type 1 diabetes [50]. Another interesting example is the *TNFRSF1A* locus (electronic supplementary material, table S1), where the same allele increases the risk of multiple sclerosis but decreases the risk of ankylosing spondylitis (AS). Intriguingly, this mimics the effect of response to anti-TNF treatment which can reduce symptoms of AS, but increased the relapse rate of multiple sclerosis in a clinical trial.

There are a number of associations shared between infectious disease and non-infectious disease, highlighting the fact that pleiotropy can potentially lead to conflicting evolutionary pressures. Specific examples include susceptibility loci for inflammatory bowel disorder (IBD) and mycobacterial infection, including at the gene *NOD2*, where a variant upstream of *NOD2* reduces risk of IBD but increases risk of leprosy. The MHC region is a classic example where HLA alleles are strongly linked to both autoimmune disease risk and susceptibility to infectious diseases. Those confirmed by GWAS include the alleles DRB1*15 (which protects from visceral leishmaniasis [51] and leprosy [52], but increases the risk of MS [17]) and HLA-B57 (which is associated with slowed HIV progression [53] but increases AS risk [54]). Beyond autoimmune disease, variants around the *APOL1* locus (electronic supplementary material, table S1) that reduce susceptibility to trypanosome infection increase the risk of two specific types of kidney disease in individuals with African ancestry [55].

One common approach to detecting pleiotropy is to compare the lists of SNPs significantly associated with each phenotype separately. This will typically underestimate the extent of pleiotropy because SNPs with real effects may happen not to reach significance for one or other phenotype (depending on the sample size). Instead, it is more natural to jointly model the effect of a SNP across different phenotypes. These model comparison approaches are better powered to detect pleiotropy and provide a better assessment of the genetic relationship between diseases [56]. They can even increase power to detect association for individual phenotypes [57].

The genetic relationship between diseases can be studied via polygenic effects as well as at individual loci. For example, a recent study of five psychiatric diseases reported significant genome-wide genetic correlations among several pairs of traits, including a genetic correlation of 0.68 for schizophrenia and bipolar disorder [58]. A more recent study of 276 genetic correlations among 24 traits with publicly available GWAS summary statistics reported significant genome-wide genetic correlations for many traits, including positive genetic correlations between schizophrenia and anorexia nervosa, and between autism and educational attainment [59].

## 6. Rare variant association studies

The genotyping arrays used in GWAS studies predominantly typed common variants. As a consequence, these studies are unable to systematically identify associations involving rare variants (including rare copy number variants), which could potentially contribute substantially to the genetic architecture of common disease [42]. A full appreciation of the genetic architecture of common disease requires direct assessment of the role of rare variants. In addition knowledge of rare risk alleles can point to potential novel therapeutic targets—even if they explain little genetic variance. This has motivated rare variant association studies, including exome sequencing and whole-genome sequencing studies. Particular sets of rare variants can also be assayed by genotyping technologies including the so-called exome arrays or genome-wide arrays with millions of markers.

The standard paradigm in rare variant association studies is to aggregate rare variants in each gene and use genes as the unit of association, via either a burden test that assumes uniform effects or an overdispersion test that allows for varying effects [60]; other biological units of association (e.g. enhancers) can also be used. Although exome and whole-genome sequencing have been extremely effective in the context of Mendelian disease, its results for common disease have been mixed, with large investments in the past few years producing a limited number of clear successes [61]. It had been argued in advance of these studies that they would probably detect rare variants of large or moderate effects on common disease, but it is becoming clear that few such variants exist with frequencies large enough for detection via this approach. However, exome sequencing studies that do not identify any individually significant genes can still be leveraged to detect and quantify polygenic signals [62], just as in GWAS [41]. An interesting strategy in the Icelandic population (for which GWAS array data are available for a substantial fraction of the population) has been to use long-range phasing of a target sample genotyped on GWAS arrays to impute variants from a small subset of sequenced individuals [63]. This approach has been particularly effective (e.g. compared with 1000 Genomes imputation studies) in identifying associations involving individual rare variants as well as aggregate gene-level associations involving sets of rare coding variants [64–66], highlighting the advantages of a close match between the reference panel and the target population when imputing rare variants.

## 7. Future directions

Over the past decade, the ability to assay genome-wide genetic variation in large numbers of individuals has led to a transformation in our knowledge of genetic variants associated with common human diseases: there are now over 10 000 published associations between DNA sequence variants and human diseases and traits. These findings have already delivered critical new insights into many of the diseases studied. For a typical disease, there will be a large number of common variants (probably thousands), each of individually small effect, that collectively explain a substantial portion of the genetic component of the disease. As existing data resources are leveraged using more sophisticated analytical approaches (e.g. analyses across diseases) and integration of different data types (e.g. functional annotation data), our understanding will grow further. Although substantial resources have already been spent on sequence-based studies of common diseases, these have as yet delivered relatively modest additional findings. As larger and better-powered sequencing studies are undertaken, knowledge of disease-associated rare variants should increase considerably. Sequencing studies are providing a more comprehensive catalogue of common and rare variants in the genome, which is informative in a number of contexts, including fine mapping.

While the pace of genetic discovery fuelled by GWAS has been extraordinary and has already delivered many important new insights into disease, systematic progress on understanding the functional mechanism of individual GWAS loci is lagging well behind their discovery. In large part, this reflects our relative lack of knowledge about and tools for unravelling gene regulation, as well as the challenge of identifying and studying the correct tissue, developmental stage and potential external stimulus. Another factor has been that groups responsible for genetic discovery are often not best suited to undertake functional follow-up. However, we are approaching a tipping point for understanding and interpreting GWAS loci. The ENCODE and Roadmap Epigenomics projects and other technological developments have greatly expanded both our background knowledge of gene regulation and the set of tools available for studying it. Methods for assessing chromatin accessibility (e.g. DNase-Seq, ATAC-seq), chromatin confirmation (e.g. 3C, 4C, 5C, Hi-C, capture-C) and the epigenetic marks associated with expression and repression of genes, together with new technologies such as the CRISPR-Cas9 genome editing system [67], now complement established genetic approaches and polygenic analyses, and offer great promise for tackling the biology underlying GWAS associations.

The next wave of genetic discovery in common diseases is likely to be driven by the growth of large population biobanks that combine genome-wide genetic information (currently genotyping arrays but in time sequence data) with extensive phenotypic information and in some cases lifestyle, diet and other environmental exposures, all measured on the same individuals (e.g. UK Biobank, a prospective study of 500 000 individuals; see Web resources). Large sample sizes are an obvious advantage of these studies for genetic discovery (recall figure 2), but they will also substantially increase our understanding of gene–gene and of gene–environment interactions. In addition, they facilitate assessment of the consequences of particular genetic variants on many phenotypes: the so-called PheWAS (phenome-wide association studies). In turn, as noted above, this offers the potential to disentangle causal relationships from epidemiological correlations using the so-called Mendelian randomization [46].

Genomics has the potential to make a significant impact on drug development pipelines. These are facing major challenges, with only a small proportion (less than 5%) of potential drugs successful in reaching the market, and estimated development costs per successful drug in the billions of dollars [68]. It has been argued that most of this failure is due to the inadequacies of preclinical models of disease, and our lack of understanding of human biology [68]. Critically, human genetic approaches provide the opportunity to learn about human biology in humans, rather than in model systems. This applies to both target discovery and target validation. First, genetic findings from both common and rare diseases can point to novel drug targets (i.e. the choice of a gene in which to intervene and the direction in which to modulate it). Second, genetics can also be used in target validation. A drug aims to intervene in human biology by altering the activity of a specific gene in a particular way, with the aim of improving a desired outcome. Genetics can be helpful here through what has been called ‘Nature's clinical trial’. To assess the therapeutic hypothesis underpinning the drug target, researchers can find individuals who, by chance, carry genetic variants whose consequences are similar to those of the drug. For example, if the drug inhibits a specific gene, one could study individuals with genetic variants which result in downregulation of the gene, or variants knocking out the function of one or both copies of the gene. If the therapeutic hypothesis is valid, these naturally occurring changes should also improve the desired outcome. Recent empirical studies have shown that the existence of supporting human genetic evidence substantially increases the chances of success for a drug [69]. This experiment can also be informative for assessing drug safety and possible side effects: if individuals carrying genetic variants that mimic the effect of the drug are at higher risk of another disease or condition, then this could point to a safety risk for the drug. Existing GWAS datasets and the growth of large population biobanks offer unprecedented opportunities to realize some of the potential for genetics to impact on drug development pipelines. As larger datasets are generated and analysed, genetics is also likely to be helpful in stratifying patient populations and moving from ‘one size fits all’ to more personalized choices of treatments.

Looking forward, genetic information will come to be collected more in clinical contexts than, as has happened to date, in research studies. Rapid decreases in costs are bringing genome sequencing closer to routine clinical care, with countries and healthcare systems already embarking on projects to sequence large numbers of patients (e.g. Genomics England and Geisinger whole exome and whole genome sequencing; see Web resources). Genome sequencing will yield clinical insights beyond what could be obtained from genotyping arrays. Whole-genome and whole-exome sequencing has had, and will continue to have, a major impact on identifying the mutations causing rare genetic conditions. As has always been the case in human genetics, these findings point directly to gene function. The consequences for common diseases of extensive sequencing will depend on the contribution of rare variants to common disease [42]. Nonetheless, integration of complementary functional information from both rare and from common diseases promises to greatly increase our understanding of human biology.

We hypothesize that within 10–15 years, there will be 1 billion individuals sequenced globally, and in many cases this information will be linked to their electronic medical records. If the challenges of data accessibility and interoperability can be overcome (e.g. Global Alliance for Genomics & Health; see Web resources), these resources will be transformative. Genetics will become an empirical big-data science: to understand the consequences of a particular mutation or the likely outcomes of different treatment choices, or to assess disease risk, sophisticated analytical tools will extrapolate from the data of genetically similar individuals under similar conditions. The progress of the past decade is often referred to as the genetic revolution. The imminence of ubiquitous genome sequencing means that a second genetic revolution is now under way.

## 8. Web resources

NHGRI GWA Catalogue: http://www.genome.gov/GWAStudies and http://www.ebi.ac.uk/fgpt/gwas/.

Haplotype Reference Consortium: http://www.haplotype-reference-consortium.org/.

UK Biobank: http://www.ukbiobank.ac.uk/.

UK Biobank documentation for genotype imputation and genetic association studies: http://biobank.ctsu.ox.ac.uk/crystal/refer.cgi?id=157020.

Genomics England: http://www.genomicsengland.co.uk/.

Geisinger whole genome sequencing: http://www.geisinger.org/for-researchers/initiatives-and-projects/pages/whole-genome-sequencing.html.

Global Alliance for Genomics & Health: http://genomicsandhealth.org/.

## Supplementary Material

Examples of biological insights about functional mechanisms gained from GWAS. References
