# Supplementary material for: Progress and promise in understanding the genetic basis of common diseases
Source: Proc Biol Sci. 2015 Dec 22;282(1821):20151684. doi: 10.1098/rspb.2015.1684 (PMC4707742; doi:10.1098/rspb.2015.1684)
Supplement: Examples of biological insights about functional mechanisms gained from GWAS. References [file rspb20151684supp1.docx]

**Table S1. Examples of biological insights about functional mechanisms gained from GWAS.** For most examples, we provide a GWAS discovery reference and a more recent reference elucidating function.

| Disease/trait | Gene, function | Causal variant(s) | References |
| --- | --- | --- | --- |
| Age-related macular degeneration | *C3*,  complement system | Coding / proteolytic CFH and CFI inactivation | Fristche et al. 2013 Nat Genet^1^  Seddon et al. 2013 Nat Genet^2^ |
| Alzheimer’s disease | *TREM2*,  microglial response | Coding / missense | Jonsson et al. 2013 NEJM^3^  Guerreiro et al. 2013 NEJM^4^ |
| Breast cancer | *FGFR2*,  oncogene | FOXA1, E2F1 binding | Hunter et al. 2007 Nat Genet^5^  Meyer et al. 2013 AJHG^6^ |
| Blond hair in Europeans | *KITLG*,  Melanocyte control | LEF1 binding | Sulem et al. 2007 Nat Genet^7^  Guenther et al. 2014 Nat Genet^8^ |
| Blond hair in  Melanesians | *TYRP1*,  Melanin synthesis | Coding | Kenny et al. 2012 Science^9^ |
| Coronary Artery Disease | 9p21 gene desert, enhancer-rich | STAT1 binding | McPherson et al. 2007 Science^10^  Harismendy et al. 2011 Nature^11^ |
| Crohn’s disease | *IRGM*,  autophagy | 20kb deletion +  miR-196 binding | McCarroll et al. 2008 Nat Genet^12^  Brest et al. 2011 Nat Genet^13^ |
| Crohn’s disease | *ATG16L1*,  autophagy | Coding / caspase cleavage motif | Barrett et al. 2008 Nat Genet^14^  Murthy et al. 2014 Nature^15^ |
| End-stage renal disease | *APOL1*, trypanosome lysing | Coding | Kao et al. 2008 Nat Genet^16^  Genovese et al. 2010 Science^17^ |
| Fetal hemoglobin (HbF) | *BCL11A*,  HbF regulation | GATA1, TAL1 binding | Menzel et al. 2007 Nat Genet^18^  Bauer et al. 2013 Science^19^ |
| LDL cholesterol | *SORT1*,  hepatic secretion | C/EBP binding | Teslovich et al. 2010 Nature^20^  Musunuru et al. 2010 Nature^21^ |
| Lupus | *TNFAIP3*,  NF-κB regulation | NF-κB binding | Graham et al. 2008 Nat Genet^22^  Adrianto et al. 2011 Nat Genet^23^ |
| Multiple sclerosis | *TNFRSF1A*,  TNF-blocking | Splice site | De Jager et al. 2009 Nat Genet^24^  Gregory et al. 2012 Nature^25^ |
| Prostate cancer | 8q24 gene desert, *MYC* oncogene | Long-range TCF7L2 binding | Pomerantz et al. 2009 Nat Genet^26^  Sur et al. 2012 Science^27^ |
| Prostate cancer | 17q24 gene desert, *SOX9* oncogene | Long-range AR, AP-1 binding | Gudmundsson et al. 2007 Nat Genet^28^  Zhang et al. 2012 Genome Res^29^ |
| Prostate cancer | *RFX6*,  oncogene | HOXB13 binding | Takata et al. 2010 Nat Genet^30^  Huang et al. 2014 Nat Genet^31^ |
| Skin, eye, hair pigmentation | *OCA2*,  melanin production | Long-range HTLF, LEF1, MITF binding | Han et al. 2008 PLoS Genet^32^  Visser et al. 2012 Genome Res^33^ |
| Type 1 diabetes | *IFIH1*,  antiviral response | Coding + splice site | Smyth et al. 2006 Nat Genet^34^  Nejentsev et al. 2009 Science^35^ |
| Type 2 diabetes | *CCND2*,  pancr. β cell mass | Regulatory (pancreatic islet cells, adipose tissue) | Morris et al. 2012 Nat Genet^36^  Steinthorsdottir et al. 2014 Nat Gen.^37^ |

**References**

1. Fritsche, L.G. *et al.* Seven new loci associated with age-related macular degeneration. *Nat Genet* **45**, 433-9, 439e1-2 (2013).

2. Seddon, J.M. *et al.* Rare variants in CFI, C3 and C9 are associated with high risk of advanced age-related macular degeneration. *Nat Genet* **45**, 1366-70 (2013).

3. Jonsson, T. *et al.* Variant of TREM2 associated with the risk of Alzheimer's disease. *N Engl J Med* **368**, 107-16 (2013).

4. Guerreiro, R. *et al.* TREM2 variants in Alzheimer's disease. *N Engl J Med* **368**, 117-27 (2013).

5. Hunter, D.J. *et al.* A genome-wide association study identifies alleles in FGFR2 associated with risk of sporadic postmenopausal breast cancer. *Nat Genet* **39**, 870-4 (2007).

6. Meyer, K.B. *et al.* Fine-scale mapping of the FGFR2 breast cancer risk locus: putative functional variants differentially bind FOXA1 and E2F1. *Am J Hum Genet* **93**, 1046-60 (2013).

7. Sulem, P. *et al.* Genetic determinants of hair, eye and skin pigmentation in Europeans. *Nat Genet* **39**, 1443-52 (2007).

8. Guenther, C.A., Tasic, B., Luo, L., Bedell, M.A. & Kingsley, D.M. A molecular basis for classic blond hair color in Europeans. *Nat Genet* **46**, 748-52 (2014).

9. Kenny, E.E. *et al.* Melanesian blond hair is caused by an amino acid change in TYRP1. *Science* **336**, 554 (2012).

10. McPherson, R. *et al.* A common allele on chromosome 9 associated with coronary heart disease. *Science* **316**, 1488-91 (2007).

11. Harismendy, O. *et al.* 9p21 DNA variants associated with coronary artery disease impair interferon-gamma signalling response. *Nature* **470**, 264-8 (2011).

12. McCarroll, S.A. *et al.* Deletion polymorphism upstream of IRGM associated with altered IRGM expression and Crohn's disease. *Nat Genet* **40**, 1107-12 (2008).

13. Brest, P. *et al.* A synonymous variant in IRGM alters a binding site for miR-196 and causes deregulation of IRGM-dependent xenophagy in Crohn's disease. *Nat Genet* **43**, 242-5 (2011).

14. Barrett, J.C. *et al.* Genome-wide association defines more than 30 distinct susceptibility loci for Crohn's disease. *Nat Genet* **40**, 955-62 (2008).

15. Murthy, A. *et al.* A Crohn's disease variant in Atg16l1 enhances its degradation by caspase 3. *Nature* **506**, 456-62 (2014).

16. Kao, W.H. *et al.* MYH9 is associated with nondiabetic end-stage renal disease in African Americans. *Nat Genet* **40**, 1185-92 (2008).

17. Genovese, G. *et al.* Association of trypanolytic ApoL1 variants with kidney disease in African Americans. *Science* **329**, 841-5 (2010).

18. Menzel, S. *et al.* A QTL influencing F cell production maps to a gene encoding a zinc-finger protein on chromosome 2p15. *Nat Genet* **39**, 1197-9 (2007).

19. Bauer, D.E. *et al.* An erythroid enhancer of BCL11A subject to genetic variation determines fetal hemoglobin level. *Science* **342**, 253-7 (2013).

20. Teslovich, T.M. *et al.* Biological, clinical and population relevance of 95 loci for blood lipids. *Nature* **466**, 707-13 (2010).

21. Musunuru, K. *et al.* From noncoding variant to phenotype via SORT1 at the 1p13 cholesterol locus. *Nature* **466**, 714-9 (2010).

22. Graham, R.R. *et al.* Genetic variants near TNFAIP3 on 6q23 are associated with systemic lupus erythematosus. *Nat Genet* **40**, 1059-61 (2008).

23. Adrianto, I. *et al.* Association of a functional variant downstream of TNFAIP3 with systemic lupus erythematosus. *Nat Genet* **43**, 253-8 (2011).

24. De Jager, P.L. *et al.* Meta-analysis of genome scans and replication identify CD6, IRF8 and TNFRSF1A as new multiple sclerosis susceptibility loci. *Nat Genet* **41**, 776-82 (2009).

25. Gregory, A.P. *et al.* TNF receptor 1 genetic risk mirrors outcome of anti-TNF therapy in multiple sclerosis. *Nature* **488**, 508-11 (2012).

26. Pomerantz, M.M. *et al.* The 8q24 cancer risk variant rs6983267 shows long-range interaction with MYC in colorectal cancer. *Nat Genet* **41**, 882-4 (2009).

27. Sur, I.K. *et al.* Mice lacking a Myc enhancer that includes human SNP rs6983267 are resistant to intestinal tumors. *Science* **338**, 1360-3 (2012).

28. Gudmundsson, J. *et al.* Two variants on chromosome 17 confer prostate cancer risk, and the one in TCF2 protects against type 2 diabetes. *Nat Genet* **39**, 977-83 (2007).

29. Zhang, X., Cowper-Sal lari, R., Bailey, S.D., Moore, J.H. & Lupien, M. Integrative functional genomics identifies an enhancer looping to the SOX9 gene disrupted by the 17q24.3 prostate cancer risk locus. *Genome Res* **22**, 1437-46 (2012).

30. Takata, R. *et al.* Genome-wide association study identifies five new susceptibility loci for prostate cancer in the Japanese population. *Nat Genet* **42**, 751-4 (2010).

31. Huang, Q. *et al.* A prostate cancer susceptibility allele at 6q22 increases RFX6 expression by modulating HOXB13 chromatin binding. *Nat Genet* **46**, 126-35 (2014).

32. Han, J. *et al.* A genome-wide association study identifies novel alleles associated with hair color and skin pigmentation. *PLoS Genet* **4**, e1000074 (2008).

33. Visser, M., Kayser, M. & Palstra, R.J. HERC2 rs12913832 modulates human pigmentation by attenuating chromatin-loop formation between a long-range enhancer and the OCA2 promoter. *Genome Res* **22**, 446-55 (2012).

34. Smyth, D.J. *et al.* A genome-wide association study of nonsynonymous SNPs identifies a type 1 diabetes locus in the interferon-induced helicase (IFIH1) region. *Nat Genet* **38**, 617-9 (2006).

35. Nejentsev, S., Walker, N., Riches, D., Egholm, M. & Todd, J.A. Rare variants of IFIH1, a gene implicated in antiviral responses, protect against type 1 diabetes. *Science* **324**, 387-9 (2009).

36. Morris, A.P. *et al.* Large-scale association analysis provides insights into the genetic architecture and pathophysiology of type 2 diabetes. *Nat Genet* **44**, 981-90 (2012).

37. Steinthorsdottir, V. *et al.* Identification of low-frequency and rare sequence variants associated with elevated or reduced risk of type 2 diabetes. *Nat Genet* **46**, 294-8 (2014).
